# “Is This the Family Path?”: Understanding the Lived Experience of Motherhood for Women Who Have Lost Their Own Mother to Suicide

**DOI:** 10.1177/00302228251317371

**Published:** 2025-01-31

**Authors:** Sara Raby, Joel R. Anderson

**Affiliations:** 1 School of Behavioural and Health Sciences, 95359 Australian Catholic University, Fitzroy, VIC, Australia; 2 110434 Australian Research Centre in Sex, Health, & Society, La Trobe University, Melbourne, VIC, Australia

**Keywords:** maternal suicide, motherhood, suicide bereavement, intergenerational suicide, interpretative phenomenological analysis

## Abstract

For daughters who have lost their mother to suicide, resurgences of grief may occur, triggered by anniversaries, family occasions, and by entering motherhood. When these women become mothers, they must adjust to the joys and stressors of motherhood, without the emotional and practical support that a maternal grandmother would often provide. This study provides an in-depth qualitative exploration of the experiences of women who have experienced their own mother suicide. Semi-structured interviews were conducted with a purposive sample of five mothers who have experienced their mother suicide. Data were collected and analysed using Interpretative Phenomenological Analysis (IPA). Analysis revealed five group experiential themes: *lacking a guiding maternal figure, navigating identity and expectations, carrying the legacy of maternal suicide, motherhood as healing,* and *the trajectory:is this the family path?*. This study identifies key challenges and specific needs of mothers who have experienced their mother suicide.

For every suicide death that occurs, there are also ‘suicide loss survivors’, people such as family or friends, who are left behind to make sense of and grieve the loss of their loved one (Maple et al., 2019). During this process, suicide loss survivors experience a range of unique challenges, including complicated grief, an increased risk of developing mental health disorders, and issues with their physical health (Allphin, 2018; Kõlves & de Leo, 2018; Shields et al., 2017). Research has shown that children of parents who have suicided are more at risk of suicide themselves (Ranning et al., 2022). Mechanisms underlying the intergenerational nature of suicide have not been clearly established, but researchers suggest that social, environmental, and genetic factors are at play (Kendler et al., 2020; O’Reilly et al., 2020). Some studies have explored the relational complications that occur for those bereaved by family suicide, including complex emotional and communication processes, and how the familial coping strategies can shape individual’s reactions to their loss (Creuzé et al., 2022; Franco, 2023; Hung & Rabin, 2009). In addition to these difficulties, suicide loss survivors frequently experience social stigma, where societal negative attitudes about death by suicide, leave survivors experiencing shame and isolation in their bereavement process (Hanschmidt et al., 2016). While bereavement is a well-established and extensively researched phenomenon that occurs immediately following the death of a loved one, some researchers propose that bereavement is not linear but more of an ongoing and cyclical process, re-experienced by the bereaved throughout their lives (Rowe & Harman, 2014).

For daughters who have lost their mother to suicide, resurgences of grief may occur, triggered by anniversaries, family occasions, and developmental milestones (Rando, 1993). One experience that could easily trigger a resurgence of grief is the transition to motherhood, an experience that is a significant life event, bringing both joy and many stressors (Lévesque, 2020). As mothers are required to adjust to new tasks and responsibilities, their daily lives are drastically changed, both physically and psychologically (Miller, 2007). Maternal grandmothers traditionally play an important role in supporting the transition to motherhood, assisting the new-mother practically and emotionally (Mitchell & Green, 2002). However, in considering that not all mothers have the support of their mother during this time, a increasing body of research has emerged, exploring the experience of women who have who have lost their own mother to death, before having their own children (Mireault et al., 2002; Walsh et al., 2023).

Maternally bereaved women with children, described as ‘motherless mothers’, have a unique experience in their journey to motherood after the loss of their own mother. Research has shown that motherless mothers experience a lack of practical and emotional support that is usually provided by the maternal grandparent (Walsh et al., 2023). Motherless mothers have been shown to be overprotective and perfectionistic, possibly in an attempt to overcompensate in the maternal role (Mireault et al., 2002). In addition, motherless mothers report persistent sadness and worry about the maternal role (Edelman, 2012). In this paper, we argue that an important task for women who have experienced their mother suicide is to re-greive the loss of their mother and re-parent themselves, as they raise their own children.

A single study has explored the specific experience of mothers who are survivors of maternal suicide loss (Leichtentritt et al., 2018). Findings from this qualitative study from Israel explain a desire for women who are survivors of maternal suicide loss to move away from their past, while simultaneous realizing they are unable to escape it. Survivors were found to have a sense of helplessness and lack of adaptive emotional responses, resulting in a tendency to suppress their feelings, remain detached, and have difficulty discussing their needs. Given that women whose mothers have suicided are at greater risk of psychosocial difficulties, including suicide, and may be limited in their supports by the social stigma experienced by suicide loss survivors, understanding their experience and needs during motherhood are of critical importance.

Therefore, the current study aimed to contribute to the body of research about women who have experienced maternal suicide loss and gain a detailed understanding of how they experience motherhood. The specific research questions were: what is the lived experience of motherhood for women who have experienced their mother suicide? and, what are their support needs during their transition to motherhood and in the early years of parenting?

## Method

### Sample and Recruitment

Advertisements were circulated on social media to suicide loss support groups. Due to the complex nature of the topic, participants were recruited via snowballing approach. The final purposive sample comprised five female participants who met criteria: (a) were over 18 years old; (b) lived in Australia; (c) had experienced their mother suicide over five years ago; (d) each had at least one child living at home at the time of the interview. Table 1 outlines sociodemographic information of participants. A small sample size is aligned with IPA methodology (Smith et al., 2009), which allows for a detailed case-by-case analysis of the participants’ experience.

**Table 1.** Sociodemographic Characteristics of Participants.

Participant pseudonym	Current age	Age at mother’s suicide	Current Relationship status	Number of children	Highest educational level
Amy	39	13	Engaged	3	Cert IV
Olivia	37	16	Single	1	Masters
Ruby	42	9	De-facto	1	Diploma
Isla	43	23	Married	3	Diploma
Chloe	35	1.5	Engaged	2	Masters

### Design

The lived experience of mothers who have experienced their own mother suicide was explored using a qualitative design, with an interpretative phenomenological approach. Interpretative Phenomenological Analysis (IPA) was selected in the proposed study, due to its ability to provide a full and detailed account of the participant’s experience and the meaning they assign to it, from their own perspective (Smith et al., 2009). Additionally, IPA is recognised as exceptionally useful when exploring issues that are complex and emotionally laden (Smith & Osborn, 2015).

### Procedure

The protocol (2024–3580H) was reviewed by the Human Research Ethics Committee at Australian Catholic University. Interested participants were provided the participant information letter via email, ensuring they were aware of the sensitive nature of the topic, that they feel comfortable discussing this, and have appropriate supports in place. Semi-structured interviews were held online, via Zoom, at a date and time according each participant’s preference. Prior to the interview, written consent was obtained via email. At the beginning of the interview, the purpose of the study, procedures, confidentiality were explained, ensuring that the participants were aware that participation is voluntary and they can withdraw at any time. Interviews were video recorded and took approximately 90 minutes. The interviews began by inviting the participant to give a detailed account of their feelings and experience of motherhood, having lost their own mother to suicide. An interview guide was used, with prompts, where needed (see Appendix), however the interview was largely conversational, directed by the participant.

After the interview, participants were debriefed and thanked for their time, ensuring they were comfortable to end the meeting. Participants were provided details to contact for further support (as per the debriefing statement). Interviews were subsequently transcribed and participants were emailed their transcript and given up to seven days to make changes or add any additional comments. All personal or identifying information were removed and names were replaced with pseudonyms, to protect participants’ identity. Data is stored in a confidential password protected University share-drive. Participants received a AUD$50 Visa gift voucher via email, for reimbursement for their time or travel and to show appreciation for their involvement in the study.

### Data Analysis

Following transcription, data were analysed using an Interpretative Phenomenological Analysis (IPA) approach. As required by IPA, a rich engagement and interpretative process of data analysis was adopted, aiming to make sense of the participant making sense of their experience (Smith, 2017). Interview transcripts were analysed by the primary researcher who has previously conducted an IPA, utilising the six-step process of IPA as recommended by Smith et al. (2009). First, the researcher read and re-read each transcript. Initial note taking began to capture observations, which was then followed by developing emerging themes for each case. The researcher then searched for connections across the developing themes for each case, repeated with each case, and finished by looking for patterns across cases to derive the final Group Experiential Themes.

### Reflective Statement

An assumption of the IPA method is that researchers are interpreting participant’s data through the lens of their own lived experience, preconceptions, and understanding of the existing literature, however, it is important that this is done with the participant’s experience at the heart of the enquiry (Engward & Goldspink, 2020). Therefore, in order to achieve this, a reflexive journal was kept throughout the study process and debriefing with the supervisor followed each participant interview. An additional step was included which involved identifying the researcher’s orientation and potential bias in discussion with the supervising researcher and throughout the reflexive journaling process.

### Positionality Statement

The researchers each have experience of family member’s suicide (although not of their mothers). The researchers team also have mixed experiences of being parents. It is understood that this lived experience could influence ideas about what participants’ experience might be. Thus, in order to ensure unjustified assumptions about participants of this study, the researchers had to remain open-minded that participants experience may greatly differ from these assumptions.

## Results

### Group Experiential Themes and Sub-Themes

Five group experiential themes and several sub themes were identified during the analysis, as represented in Figure 1, and indicated in Table 2. The first theme describes how participants experienced what was described as “a lack of a guiding maternal figure”. The second theme explores how participants had to navigate their changing identity, pressure and expectations. In the third theme we discuss how participants carried a unique legacy of surviving their mother’s suicide. The fourth theme details how the participants experienced motherhood as a healing journey. And finally, the fifth theme examines the permeating question that participants had about whether suicide was an uncontrollable trajectory within their family system. These themes are detailed in the sections below using illustrative excerpts, representing a small sample of the rich and detailed accounts by participants.

**Figure 1.**
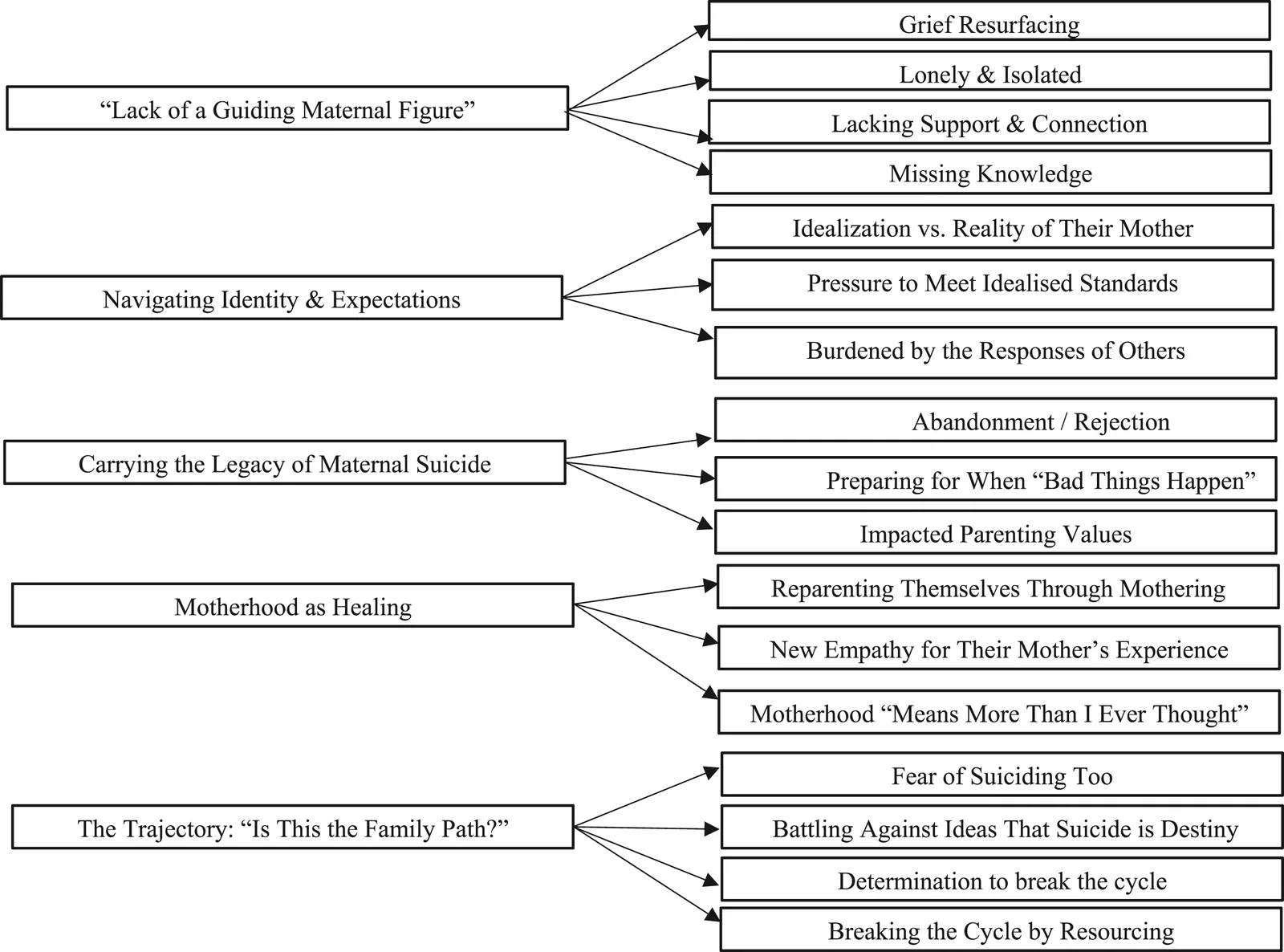
Pictorial representation of group experiential themes and subthemes.

**Table 2.** Prevalence of Group and Personal Experiential Themes by Each Participant (Pseudonym).

Group/Personal experiential themes	Amy	Olivia	Ruby	Isla	Chloe
“Lack of a guiding maternal Figure”					
Grief resurfacing	√	√	√	√	√
Lonely & Isolated	√	√	√	√	
Lacking support & connection	√	√	√	√	√
Missing knowledge	√	√	√		√
Navigating identity & expectations					
Idealization versus Reality of their mother	√		√		√
Pressure to meet idealised standards	√		√		
Burdened by the responses of others	√	√			√
Carrying the legacy of maternal suicide					
Abandonment / Rejection	√	√	√	√	
Preparing for when “bad things happen”	√	√			
Impacted parenting values	√	√		√	
Motherhood as a healing Journey
Reparenting themselves through mothering		√	√	√	√
New empathy for their Mother’s experience	√	√	√	√	√
Motherhood “means more than I ever thought”			√	√	√
The trajectory: “Is this the family path?”					
Fear of suiciding too	√	√	√		√
Battling Against ideas that suicide is destiny	√	√	√		√
Determination to break the cycle	√	√	√	√	√
Breaking the cycle by resourcing	√	√	√	√	√

#### “Lack of a Guiding Maternal Figure”

Traditionally, the ‘mother’ figure plays an important role for a child’s growth and development, including teaching them about the world, nurturing physical and emotional needs, providing protection and love, and supporting them to develop a sense of self. Participants reflected on what not having ‘a mother’ figure was like for them.

##### Grief Resurfacing

All participants experienced a reappearance of grief and longing for their mother when they had their own children. Some participants found watching other mothers receiving the support of their mother during the postpartum period to be triggering, Olivia explained: “*I think it's hard when you watch others getting that experience with their mums. I felt like it did retrigger grief. Quite profoundly… I just wish she was here. I wish she could meet my child*”. On the other hand, Chloe and Isla found looking at their children to be the trigger:

I look at my daughter and I think, she's the same age as what my sister was [when my mum died]. But she's such a baby…they’re still so little. It definitely comes in waves. (Isla)

Every time I fed my son, or I was looking at him... I was like, oh my gosh, this is the time I would have spent with mum. (Chloe)

##### Lonely and Isolated

All participants spoke about the significant loneliness and isolation they felt after becoming a mother. Isla reported feeling very lonely and thinking “*I'm doing it a lot by myself*”. Amy reflected on the unique feeling of isolation that she felt from not knowing anyone that understood what it was like experiencing maternal suicide:

If they die in a different way, you've then usually got people that can understand what you're going through and relate to you. I found… even clinicians, they haven't had this experience. They don't know… It feels very lonely.

##### Lacking Support and Connection

All participants spoke about lacking practical and emotional support that the maternal grandparent would usually provide after a woman has a child. Olivia explained her sense of lacking support and connection was triggered when she attended her local ‘mother’s group’, stating*: “they've all got their mothers and their mums are giving them tips. And their mum comes into the house and helps them out. And you don't have any of that*”. Amy echoed the sense of lacking support during early motherhood, saying “*nobody's bringing me lasagnas or whatever it is, or practical help, that doesn't exist*”. While Chloe experienced others that filled that role, she highlighted the uniqueness of what ‘a mother’ would bring: “*a mum would just kind of get it a bit more, and also know what you might need, and maybe anticipate your needs*”.

##### Missing Knowledge

Most participants acknowledged missing knowledge and guidance, about the world, practically and socially, Ruby stated: “*it’s taken me quite a while to figure out, to actually understand, how the world works*”. The participants spoke about how they felt they were missing the knowledge about how to parent their child, that would typically be passed down from one’s own mother. Olivia reflected on how “*there was information that should have been passed on to me that I didn’t get*”. She explained an example of how she was hesitant to teach her daughter about the Easter Bunny or Santa Claus, because she wanted to be honest with her about life, but also reflected that:

There was no one passing down social norms …I was just making it up as I go… and what felt right to me was to be in integrity with my child. Rather than following traditions, because I didn't have the modelling of the traditions.

Amy also experienced this feeling of missing important practical information about life that is also needed for parenting:

I had no idea you need a GP appointment to get a specialist appointment or just any of these basic rules. No clue about any of it.…not even knowing what help is available or not even knowing how to access certain things.

Amy reflected on how she used the internet to fill this gap, explaining “*The internet is my parent*”. Chloe had similar sentiments: “*My Google search history was out of control, because … I had no idea what was going on*”.

#### Navigating Identity and Expectations

Participants reflected on the image they had of their mother, while trying to make sense of their own identity as a mother. They noticed high standards they set for themselves and some felt the pressure of others’ responses.

##### Idealisation versus Reality of Their Mother

Some participants spoke about how after their mother’s death, people spoke about her in an idealized way, Chloe stated, “*everyone presented mum in this positive light, we never talked about like any of the deeper stuff, any of the harder stuff, any of the negative stuff about her*”. This left participants’ feeling confused and lacking a balanced understanding of who their mother was:

Mum was kind of glorified after her death…like she was such a beautiful lady, but that wasn't obviously always who she was…. when she died and everyone talked about her like she was a Saint, like she didn't have any faults… it was very confusing to me. (Ruby)

##### Pressure To Meet Idealized Standards

Children have a natural tendency to identify with their same-sex parent. So, because of the way their mother was idealized, participants felt a subsequent pressure to live up to the same image. Ruby explained that she thought, *“that’s what I'm supposed to be, that’s what everyone says Mum is. Mum's perfect!”*. Amy also felt the same pressure, saying that her mother was deserving of being put on a pedestal but that, *“when someone's gone, they don't have any flaws at all in a way. It puts an impossible pressure on yourself to live up to that.*”.

##### Burdened By Responses of Others

In navigating the postpartum period, most participants had people who were concerned for their wellbeing, but found that their responses were burdensome. Chloe explained:

I sensed everyone being extra worried about me. It was like they were almost projecting their worries onto me. I felt burdened by their emotions…. Everyone was so clouded by their own perception of what was gonna happen, that I got lost.

Similarly, Olivia experienced what she described as a stigmatising experience with her therapist, explaining, “[she] was always on the watch, [saying], do you think you've got postpartum depression? which felt stigmatizing as I wasn’t showing signs of depression at that time.”

For Ruby, who experienced suicidal ideation during the postpartum period, it was staff at the hospital she was staying that provided statistics in an attempt to persuade her away from suicide:

One of the ladies said to me…your chances of suiciding are that much higher because you just see it as an option, like, you can't do that to your son, kind of thing.

She explained that, despite knowing the familial risk and knowing that her son was her reason for living, she “*was so disconnected then, that [message] just wasn't getting in*.”

#### Carrying the Legacy of Maternal Suicide

Experiencing their mother suicide left participants having to process difficult feelings. They also found their behaviour, values and parenting style were impacted by the experience.

##### Abandonment/Rejection

Most participants spoke about feelings of abandonment and rejection when reflecting on their mother’s suicide. Olivia reflected on how the abandonment she felt from her mother’s suicide left her thinking “*oh, people just disappear anyway*”. For Amy, the abandonment and rejection she felt left her with strong feelings of unworthiness. She explains the message she received from her mother’s suicide was “*that I wasn't enough to live for, or worthy of love, or enjoyable to stick around for. That's probably putting it pretty mildly, considering that it was enough for her to kill herself and abandon me permanently*.”

##### Preparing for When “Bad Things Happen”

For some participants, the experience of their mother’s suicide left them with an understanding that ‘bad things happen’ in life and felt this more strongly when their children were born. Olivia described that she parents her child to be independent, explaining that “*I want to make sure she's got skills to look after herself…I really empower her and I think that's because I've had to be highly self-reliant as well*”. She went further to say that sometimes:

I look for signs to see whether she needs me, or whether, if I left, would she be, okay? I do find myself scoping for those signs, like, does this kid need me or would she be okay if I didn't exist?

Despite reporting that she no longer experiences suicidal ideation since having children, Amy also felt an ongoing need to prepare her family, explaining that:

I sometimes can be really insistent on pushing my partner to be very capable and equal around the house… because in the back of my mind, I'm always preparing for a scenario where he is left to parent alone. I try to make myself as replaceable as possible, to make it less painful or disruptive for them, because my world and life was shattered, so I try to protect them from that.

##### Impacted Parenting Values

Most of the participants reported impacted parenting values as a result of their mother’s suicide, in varying ways. Olivia said that the experience has “*made me more driven and more protective. I'm quite a fierce mother bear*.” Similarly, Isla reported being more protective and “*more aware of my children's mental health*”.

Amy described that the main message she received from her mother’s suicide was that “*life’s not worth living*”, and as such, she worked hard to teach her children the importance of life:

If you're bringing somebody into the world, you have to be able to tell them why life is worth living and why it's great... You need to have a strong reason. I think that is something that I've had to develop myself over a lot of time.

#### Motherhood as Healing

All of the participants spoke about ways they experienced motherhood as healing, whether it was through acknowledging their own needs, connecting with their mother’s suicide with compassion, or experiencing a special purpose to motherhood.

##### Re-Parenting Themselves through Mothering

Despite lacking the ‘mother figure’, the participants reflected on coming to learn to mother themselves, and how important this was in parenting their own children. Ruby explained “*You've got to make sure that you’re mothering you in every aspect, because if you're not doing that, you don't have the flow on from that to give to someone else*”. Isla talked about taking her of her own needs and acknowledged “*If I can make myself happier and healthier and in a better place than everyone else will be too*”. Olivia acknowledged that this is an ongoing process and reflected, “*I definitely still struggle to meet my own emotional needs*”.

##### New Empathy for Their Mother’s Experience

All participants spoke about how motherhood brought them a new sense of empathy and compassion for their mothers’ suicide, Isla stated “*I can see where… those thoughts may get to a point where they are so dark that you just don't know what else to do*.” Chloe and Ruby similarly explained how motherhood brought them a new sense of understanding:

I [now] see from the inside of mothering, the struggle that being a mother can bring up, and the difficulty of it. And without support and understanding, and places to go... how much that can lead to turmoil. (Chloe)

After becoming a mother, there was more understanding of the challenges that motherhood brings and how this might have impacted their mother’s mental health, as Ruby highlights:

If we already haven’t healed from past trauma, Motherhood brings a whole range of new pictures and ideals that we need to live up to, and it just sank me. I realise now that’s what likely went on for Mum too.

##### Motherhood “Means More Than I Ever Thought”

Most participants referred to their experience of motherhood and carrying special meaning and purpose. For Ruby, motherhood was her reason for living, she stated:

When I was in hospital, [my baby] was the only thing that I felt I had to live for... We'd go for walks.... It was so exquisite and so magical having him in my life.

For Chloe, motherhood carried a special meaning of understanding what having a mother is like, through parenting her children, she stated “*I didn't get to experience what a mother was as a child and growing up. So, now, it's almost like, I'm finally getting to experience what it is…*.”. Isla also found special meaning and purpose, as she looked forward to being a mother for her children when they have children, she said “*I guess not having my mum there, I really want to be there for my own [children], if they have children, I want to give them what I didn't have*”.

#### The Trajectory: “Is This the Family Path?”

All participants experienced an underlying fear and question about the intergenerational nature of suicide within their family.

##### Fear of Suiciding Too

Most participants explained a fear that they would also meet the same fate as their mother, they feared and wondered whether they would suicide once they had children:

I saw really clearly this pattern of onset of mental health issues following, whether it was a traumatic birth or [motherhood]... since my mum, my auntie and my sister became a mother, they became unwell… and so I was pretty emotional, and I think when you have a parent that suffers from mental health… I always, growing up, had this fear that I was going to lose my mind. (Chloe)

Underlying their fear, was a sense of uncertainty about their situation. For Olivia, this fear was increased due to having her grandmother also experience suicide, she explains:

Because it's known to me that my mum's mum also committed suicide, there's this.. Transgenerational type of thing going on… I carried this paranoia, which became more pronounced after becoming a mother of a daughter as well, that I would inevitably meet the same fate… I've even found myself contemplating, would it be better to suicide while my child's younger or wait until they're older?

##### Battling Against Ideas That Suicide is Destiny

Underlying participant’s personal predisposition toward suicide, was a question they asked themselves, do I have a choice or is this biological? This was felt and described by Olivia as a “*spiritual battle between destiny and free will*”. Participants experienced an internal battle, fluctuating between feeling powerless over the familial risk of suicide, to feeling like they have personal power and a choice about their behaviour and therefore their future.

The participants spoke about needing to come to terms with two contradicting explanations for their mothers’ suicide; the side that explains their mother’s suicide as an illness, a mental health condition, a decision made without judgement, versus the explanation that she did have a choice in ending her own life. Chloe described the process as “*seeing the realness in her… accepting that what she did could have been done in full comprehension, or because she was not well, it could have been in a complete delusion*”. Amy explained that either way, there was a difficult truth to come to terms with, stating “*if she had control, then she chose to leave… That's something very painful that you have to live with. But if she was out of control, then that means you could be out of control too*”.

##### Determination to Break the Cycle

All participants spoke strongly about wanting to stop the cycle of intergenerational suicide from happening in their family. Chloe described going from fearing suicide to feeling empowered, thinking “*no, I have power in this*”. Amy spoke about how her determination came out during her postpartum period:

I look back now at [what was] probably postpartum depression... But I didn't realize it at the time because I was determined not to have it. I was like, I'm not having it. I'm not going to get it. I'm just going to get on with it.

The participants spoke about how they kept her children in mind to overcome their fears of suicide, Olivia explains: “*I've got to break the chain and not let my child go through what I've gone through*”.

##### Breaking the Cycle by Resourcing

All participants spoke about identifying the need to resource themselves to protect their mental health after they had children. Participants reflected on their mother’s life and traits to try to resource themselves with what she might have been missing, Ruby stated:

If I didn’t want to go down a similar trajectory to her, I needed to look at what happened to her, how she ended up there, what she needed to not end up there. And what do I need to not end up there? … [I noticed] the patterns or the tendencies within her to withdraw, to bottle her feelings, to not deal with feelings that were there, to not express.

Participants also spoke about accessing therapy, researching online, and building their supportive community as important ways to safeguard their mental health. Chloe explained:

I was doing everything I can to come out the other side well…I really dived into making sure I was resourcing myself as much as possible.

## Discussion

The purpose of this study was to gain an understanding of the lived experience of motherhood for women who experience their mother’s suicide. The current study provided a detailed exploration of five participants experiences of motherhood, having lost their mother to suicide. Participants were found to experience difficulties relating to a lack of a guiding, maternal figure to support them, resurfacing grief, loneliness and isolation, and a lack of support that is usually provided by the maternal grandparent. This is consistent with previous research on women who have experienced their own mother’s death (Shields et al., 2017), as well as the single study that has examined maternal suicide survivor’s experience of motherhood (Leichtentritt et al., 2018). The findings go further to highlight that maternal suicide loss survivors miss practical knowledge that is usually passed down from mother to daughter in their transition to motherhood, as well as a lack of connection that ‘a mother’ figure usually brings.

Where previous resarch on motherless mothers found women to be overprotective and prefectionistic, suggesting a possible explanation of feeling the need to overcompensate in the maternal role (Mireault et al., 2002), the current study presents a different explanation for women who have survived their mothers’ suicide. Findings revealed that these women may face pressure to live up to unrealistic standards from not having a balanced understanding or memory of their mother after her death. Experiencing others’ tendency to discuss only the positive aspects of their mother, and avoidance of discussing the more challenging, darker sides, left participants with a confusing and unbalanced understanding of who their mother was, and subsequent pressure to live up to the perceptions of her idealised image. This is somewhat similar to previous research that has found suicide loss survivors experience relational complications, in emotional and communicative processes in their support systems (Hung & Rabin, 2009). Participants were found to be impacted by others responses to their predisposition to suicide, where they felt pressure and stigma, during an already vulnerable time.

Similar to previous research on suicide bereavement (Allphin, 2018; Shields et al., 2017), the current study also found that women who have lost their mother to suicide experience complicated emotions such as abandonment, rejection and anxiety, but highlights that this resurfaces during motherhood. Interestingly, the current findings go further to suggest how these feelings may impact mothers through the need to prepare their family for the case that ‘something bad’ happens, such as their own suicide. Participants described promoting independence (in children) and the development of parenting skills (in partners), so as to not have their family experience the difficulties that they faced when they lost their mother. The results of the current study strongly imply that having a mother suicide impacts one’s own parenting.

Positive impacts to parenting were also reported by participants, suggesting a unique strength and capacity for women to use their experience for growth and healing during their experience of motherhood. These findings reinforce and build on previous research that shows how bereavement following suicide loss evolves over time and can include elements of post-traumatic growth (Creuzé et al., 2022). Participants of the current study experienced motherhood as a healing journey, found a new sense of empathy and compassion for their mother’s suicide, arguably supporting them to process their resurfaced grief. Women were also found to recognise the importance of reparenting themselves and found special meaning in motherhood, indicating their own experience of post-traumatic growth.

Finally, the most compelling finding of the present study was the detailed understanding of how participants experienced a pervasive fear, uncertainty, and dissonance around their personal disposition to suicide. Where previous research has shown that women who have experienced maternal suicide loss struggle to escape their past (Leichtentritt et al., 2018), the current study provided more detailed exploration of this phenomena. Participants felt their own risk for suicide as a familial trajectory and an unanswerable question of nature versus nurture – would they suicide too? Or do they have control? There was a complex process of coming to terms with the conflict surrounding their mother’s death. Participants described, on the one hand, needing to accept that their mother may have been mentally unwell, and therefore had limited control over her decisions, but that this understanding may mean they also may become unwell during motherhood and follow a similar path of suicide. Participants also described that on the other hand, they needed to accept that there was a chance their mother had some control and choice in ending her own life, and face the pain of rejection and abandonment that this understanding brings. This side of the explanation did have protective impacts to their experience of motherhood, as they felt empowered by also ‘having a choice’ to instead do things differently.

### Limitations and Implications

#### Considerations for Interpreting the Findings

There are at least two limitations worth considering when interpreting the findings of this study. A first consideration is that there was significant variation among participant’s ages at the time of their mother’s suicide, ranging from as young as 1.5 years–23 years (although all now adults aged between 35 and 43). This variation raises critical questions about how the developmental stage at the time of loss influences memory and the perceived impact of losing a mother to suicide. For those who experienced the loss in early childhood, *childhood amnesia* likely influences direct recollections of their mother or the events surrounding her death (i.e., the relative absence of accurate autobiographical memories in the earlier years of life, see Baeur, 2015). It is worth considering that all participants – but particularly those who were younger at the time of their mother’s death – will likely have had their memories and experiences of the loss may be shaped by family narratives or external cues, increasing the potential for misinformation effects or source amnesia (in which details are non-consciously reconstructed or attributed to second-hand accounts, see Nicoladis et al., 2022).

For participants who were older at the time of their mother’s death, memory biases such as the *peak-end rule* may be particularly salient – this rule is a psychological heuristic that describes how people tend to judge an experience largely based on its most intense point (the “peak”) and its ending, rather than on the broader experience. The emotional intensity of losing a mother to suicide likely solidified vivid memories of the loss itself, while other aspects of their relationship may be less distinctly remembered (see Horwitz et al., 2024). These participants might also experience a negativity bias (focusing on the traumatic and painful aspects of the loss), or a ‘rosy retrospection’ (emphasizing positive memories of their mother while minimizing conflicts or challenges in the relationship; Zurbriggen et al., 2021).

Finally, the longer interval between the loss and the participants’ current age introduces complexity. Over time, a self-consistency bias may shape how participants integrate the experience of their mother’s suicide into their broader life narrative. This bias is the tendency for individuals to recall past events, attitudes, or behaviours in a way that aligns with their current self-concept (Conway et al., 2004). This bias arises because people are motivated to maintain a coherent and stable sense of self over time, and as a result, they may unconsciously modify or reinterpret memories to fit their current identity or worldview. In the context of this study, participants who view their mother’s suicide as a formative event might unconsciously emphasize memories that align with this interpretation. Conversely, participants who have processed their grief or engaged in therapeutic interventions might recall the event with greater nuance, balancing traumatic memories with reflections on resilience and healing. Given the stigma surrounding suicide, participants’ memories may also be influenced by societal and familial narratives about their mother’s death. This could amplify certain emotions, such as shame or isolation, or alter how participants reconstruct the loss in relation to their broader identity and experiences. Future research could focus on understanding how these memory processes operate across different ages of loss to providee insights into the interplay between developmental factors, trauma, and long-term memory reconstruction in the context of maternal suicide.

A second consideration is about the extent to which the identified themes are unique to women who have lost their mothers to suicide. Some of the themes – such as the ‘*carrying the legacy of maternal suicide*’ and the ‘*the trajectory: ‘is this the family path?*’ themes (along with a range of other subthemes) seem very specific to maternal suicide, but we acknowledge that some of these themes are likely to overlap with other experiences of suicide – including the experiences of women who lost their mothers by other causes. These themes might indeed be relevant to mother (or parent) loss of any cause. Further research is warranted to better understand the shared and distinct components of experiencing maternal suicide.

#### Research Implications

These findings suggest that women who have experienced their mother suicide experience unique challenges during motherhood, thus there is also a need for research that examines the experiences of women’s loss of a father to suicide, or a father’s loss of either parent to suicide, and subsequent impacts to their transition to parenthood. Additionally, much work remains to be done before a full understanding of the intergenerational nature of suicide is established. However, further research into the underlying mechanisms and moderators of this is recommended, with consideration to influential relational dynamics and the explanatory model used by suicide survivors in processing their loss and subsequent impacts to their locus of control.

#### Clinical Implications

Despite these limitations, the results from the current study suggest several theoretical and practical implications. Firstly, given the complex challenges that women face having lost their mother to suicide, screening for maternal suicide loss in pregnancy or postpartum clinical settings is recommended. Subsequently, intervention focussed on supporting women to understand and process the internal conflict around their mother’s death and their own predisposition to suicide, is strongly recommended. Additional supports targeted to resourcing women with knowledge about parenting, adaptive practical and emotional coping skills would be helpful, to support women to manage their own wellbeing during motherhood, despite their genetic risk of suicide.

Given the strength of the reports of the internal conflict women faced in processing their mothers suicide and what that meant for them now as mothers, a conceptual model is proposed (see Figure 2) as framework for understanding their experience. This model is presented using two halves of circles, because it is seen that both sides of the circle are needed and important to be worked through therapeutically. Clinicians may use this model to support their understanding of the lived experience of mothers who have experienced their own mother’s suicide and the emotional tasks they may require support with during motherhood.

**Figure 2.**
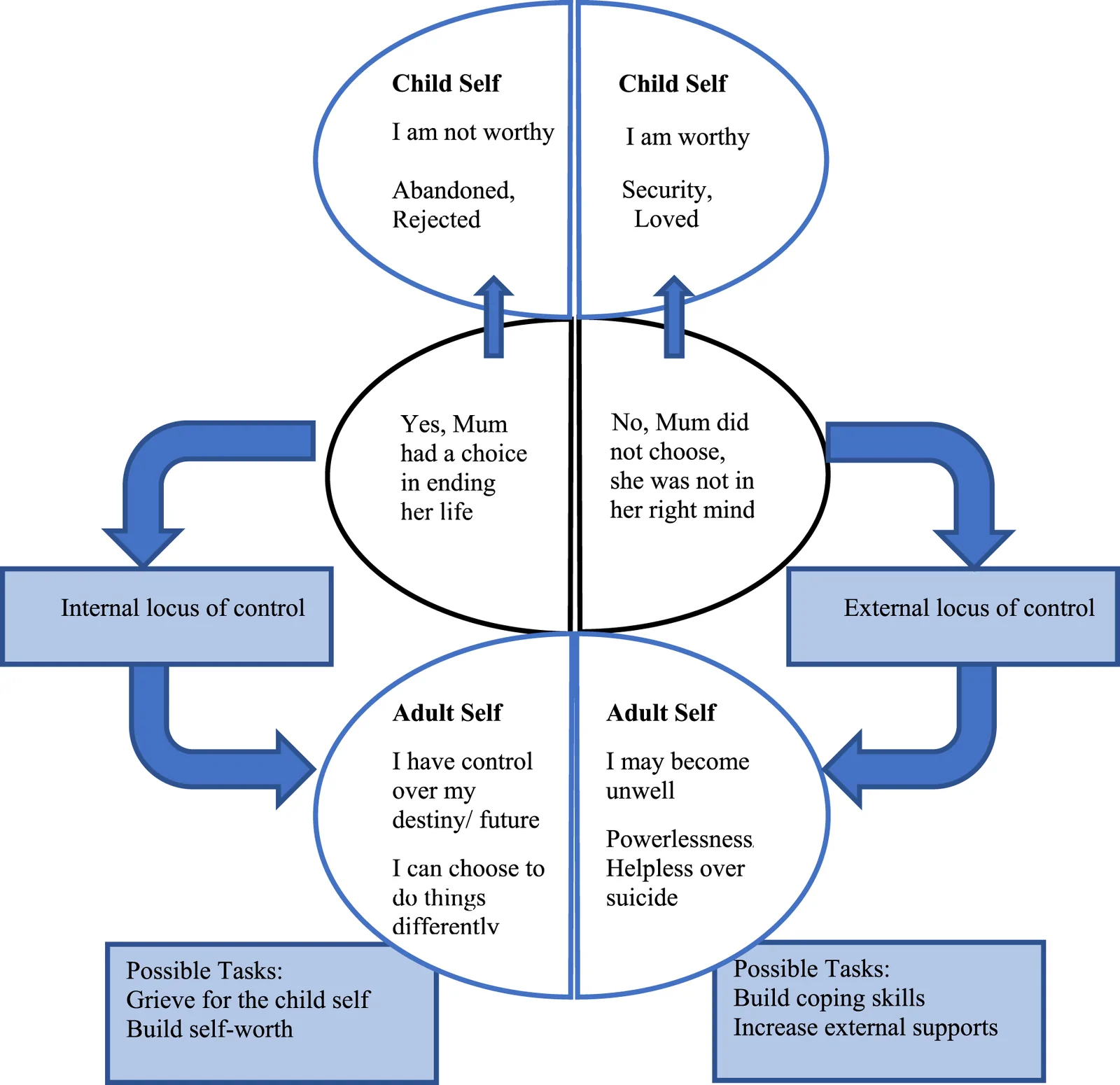
Internal conflict faced by women who have lost their mothers to suicide.

### Conclusion

No single theory, model, or study can fully explain the impacts of losing a loved one to suicide. However, this study has sought to provide a detailed understanding of the experience of motherhood for women who have lost their mother to suicide. Interpretative phenomenological analysis was used to explore the motherhood experiences of five women who have experienced their mother’s suicide. Findings suggest that these women experience a lack of a guiding maternal figure, resurfacing of grief, isolation, missing knowledge, and challenges relating to understanding their own identity as a mother. Participants felt feelings of abandonment, rejection, and a need to prepare their children or family in the case that suicide happened for them too. Participants reported a strong internal conflict in processing their own predisposition to suicide. Despite the challenges, participants found motherhood to be a healing experience, where they could reparent themselves, connect with their mother’s suicide with compassion, and experience a special meaning and purpose to motherhood. Notably, women were determined to break the cycle of potential intergeneration suicide, and found benefits in resourcing themselves to manage their own emotional wellbeing.

## Supplemental Material

Supplemental Material - “Is This the Family Path?”: Understanding the Lived Experience of Motherhood for Women Who Have Lost Their Own to Mother Suicide Supplemental Material for “Is This the Family Path?”: Understanding the Lived Experience of Motherhood for Women Who Have Lost Their Own to Mother Suicide by Sara Raby and Joel R. Anderson in Journal of Death and Dying
