# Supplementary material for: “Is This the Family Path?”: Understanding the Lived Experience of Motherhood for Women Who Have Lost Their Own Mother to Suicide
Source: Omega (Westport). 2025 Jan 31;94(1):682–701. doi: 10.1177/00302228251317371 (PMC13646440; doi:10.1177/00302228251317371)
Supplement: Supplementary file 1 [file sj-pdf-1-ome-10.1177_00302228251317371.pdf]

Appendix: Interview Guide

Opening statement

- I would like to hear about your experience of becoming a mother as a survivor of maternal suicide loss. Please tell me as much as you can about that.

Questions to be addressed via probes, if required

- Please, describe your experience of becoming a mother? Probe: Was this consistent with your expectations?
- What does it mean for you to be a mother having lost your own mother to suicide?
- What are the main challenges you have faced in early motherhood while being a survivor of maternal suicide loss? Probe: what did or what do you think would have helped you to overcome/cope with these challenges?
- What were your main worries about becoming a mother having lost your mother to suicide?
- What some of the main needs you identify, being a mother having experience maternal suicide loss?
- What are your thoughts about your own future and your child's future?
- Can you please describe your experience and relationships with your family, friends, or other supports while you were transitioning to motherhood and/or parenting your children as a survivor of maternal suicide?

Demographic questions for the conclusion of the interview

- Age
- Ethnicity
- Marital/relationship status
- How many children do you have? Age/s
- What age were you when your mother died?
- What is your highest level of education/training?
- What is your most recent employment history?
